# Retroactive interference model of forgetting

**DOI:** 10.1186/s13408-021-00102-6

**Published:** 2021-01-23

**Authors:** Antonios Georgiou, Mikhail Katkov, Misha Tsodyks

**Affiliations:** 1 Department of Neurobiology, Weizmann Institute of Science, Rehovot, Israel; 2 Department of Natural Sciences, Institute for Advanced Study, Princeton, NJ USA

**Keywords:** Theory, Memory, Retention curve, Recognition, Memory valence

## Abstract

Memory and forgetting constitute two sides of the same coin, and although the first has been extensively investigated, the latter is often overlooked. A possible approach to better understand forgetting is to develop phenomenological models that implement its putative mechanisms in the most elementary way possible, and then experimentally test the theoretical predictions of these models. One such mechanism proposed in previous studies is retrograde interference, stating that a memory can be erased due to subsequently acquired memories. In the current contribution, we hypothesize that retrograde erasure is controlled by the relevant “importance” measures such that more important memories eliminate less important ones acquired earlier. We show that some versions of the resulting mathematical model are broadly compatible with the previously reported power-law forgetting time course and match well the results of our recognition experiments with long, randomly assembled streams of words.

## Introduction

Memory has been often associated solely with the property of persistence, that is, the ability to retain and retrieve information with the passage of time. However, another equally important characteristic of memory is transience or, in other words, the ability to forget and discard information that could be no longer relevant. This process is considered crucial for memory, and it is hypothesized to be essential for adaptive behavior [1]. Traditionally, since Ebbinghaus’s seminal study [2], forgetting has been described using the retention curve. This curve is a continuous function of time $R(\tau )$, which denotes the probability that a memory of age *τ* still exists (i.e. not yet forgotten). The shape of the retaining function has been investigated through the examination of experimental data over the last century with the hope that its mathematical form will help to reveal forgetting mechanisms. Most of the studies conclude that forgetting is well described by the power-law decay [3–7]:

1 $$ R(\tau )\propto \tau ^{-\alpha }. $$

Estimates of the exponent *α* vary between studies but generally are observed to stay above 0.1, with at least one study reporting that *α* increases with the passage of time from ≈0.1 to ≈0.5 [8]. Conversely, we can define a *forgetting* rate function $F(\tau )$, expressing the probability that an available memory of age *τ* will be forgotten within the next time interval *dt* (probability of forgetting is $F(\tau )\, dt$ as $dt\rightarrow 0$). The two functions are related under the equation (see e.g. [9])

2 $$ R(\tau )= e^{-\int _{0}^{\tau }F(t)\, dt}. $$

To derive this equation, we consider a non-homogeneous Poisson point process with time-dependent rate $F(t)$ corresponding to forgetting rate defined above. The probability for a memory to still exist at time *τ* after acquisition equals the probability that no Poisson events occur in the interval $[0,\tau ]$. The probability to have *n* events in this interval is given by the Poisson distribution (see e.g. [10])

3 $$\begin{aligned}& P(n)=\frac{\Lambda ^{n}}{n!}e^{-\Lambda }, \\& \Lambda = \int _{0}^{\tau }F(t)\, dt. \end{aligned}$$

Substituting $n=0$ into this equation results in equation (2).

Equation (2) can be inverted to

4 $$ F(\tau )=-\frac{R'(\tau )}{R(\tau )}. $$

This equation means that whereas the retention function is decreasing with time (if we assume that extinguished memories cannot be reinstated), the forgetting function can in principle be both decreasing and increasing, depending on the decay speed of the retention function. In particular, an exponential retention function is a borderline case, which results in a forgetting function that is independent of time, that is, all memories have the same probability to be forgotten, irrespective of their age.

Substituting (1) into (4), we get that for power-law decay of retention, the forgetting rate will decay in time at an inversely proportional manner, regardless of the value of the exponent *α*:

5 $$ F(\tau )\propto \frac{\alpha }{\tau }. $$

In other words, somewhat paradoxically, memories that are older are more resilient (have lower probability to be forgotten at any given moment). It is important to mention that power-law forgetting can be an artifact of averaging over multiple subjects such that for each individual subject, the retention function exhibits exponential decay with time (see [11–13]), even though a study by [14] argues that even retention curves of individual subjects are still better described by power-law functions. In this study, we assume that power-law forgetting is a genuine phenomenon and further discuss this issue.

The interest in mathematical forms of memory curves was encouraged by the hope that they may shed light into the mechanisms of remembering and forgetting, which remain a mystery. Since there is no clear understanding of processes leading to forgetting, authors frequently compared many different functional forms [4, 5, 7]. Some authors went to the extreme of comparing the goodness of fit for 105 functions to 210 data sets [6]. We believe that just comparing different functional forms does not lead too far in understanding the mechanisms of forgetting. One possible alternative is to propose theoretical models implementing specific mechanisms of forgetting, ideally leading to testable experimental predictions in broad experimental settings, which potentially could more precisely constrain the mechanisms of forgetting. Mechanisms that are usually considered in relation to forgetting are passive decay of memories, interference, and consolidation (see e.g. [15]). Decay theories state that memories are degraded with time and are completely forgotten when a threshold is reached. On the other hand, the more popular interference theories suggest that prior (proactive) or subsequent (retroactive) learning disrupts memory consolidation and therefore memories are forgotten (for a review of both cases, see [15]).

A simple and elegant mathematical model of the first type is presented in [12]. Whereas it appeared that passive decay of memory strength should result in new memories gradually replacing the older ones, Kahana and Adler showed that when new memories are characterized by variable initial strengths and decay rates and are forgotten when the strength dips below threshold, the retention function converges to $1/\tau $ scaling in the limit of large *τ*. It is important that the necessary condition for this property is that the distribution of decay rates extends all the way toward zero, that is, some memories do not decay with time. For example, consider the simplest version of the model when each memory is characterized by linearly decaying strength $S(t) = a - bt$ with positive coefficients *a* (initial strength) and *b* (decay rate) chosen randomly for different memories. When the memory strength decays to zero, it is erased (forgotten). We can show that asymptotic scaling for the probability that a memory is still available at time *τ* after acquisition is given by

6 $$ R(\tau ) \approx \frac{P_{b}(0)}{\tau }\langle a\rangle , $$

where $P_{b}(b)$ is the probability density of the decay rate *b*, and $\langle a\rangle$ is the average value of the initial memory strength (see Appendix for a derivation). The condition that $P_{b}(0)>0$ also means that the average life-span of a memory is infinite. This study to a large extent demystifies the power-law scaling of retention curves; however, the assumption about the passive decay of memories does not take into account the well-documented effect of memory interference [16]. An alternative model that combines passive decay and interference was proposed in [17], where memories are characterized by a ratio of times since their acquisition to that of other memories. Recall probability in this model is assumed to depend on its “distinctiveness”, defined as an inverse of acquisition time ratios averaged over all other memories. On one hand, interference is involved since different memories interact to determine their distinctiveness; on the other hand, when time passes without any new memories being acquired, distinctiveness of all memories, and hence their recollection, declines, indicating that passive decay is also effectively at play. The authors show that this mathematical model accounts for experimental retention curves and other well-known phenomena in the recall literature, such as recency-to-primacy gradient. However, this model also rests on several strong assumptions; for example, it assumes that the time since the acquisition of each memory has to be explicitly encoded in memory.

In the current contribution, we aim at a forgetting mechanism that would be compatible with realistic retention curves, contain as few assumptions as possible, and could have a clear functional interpretation. To this end, we propose a family of phenomenological models that parallel the concept of retroactive interference and capture the statistical properties of forgetting that were previously discussed. Similarly to [12], we simplify the memory retention as a binary process (available/forgotten) and introduce the crucial notion of memory strength, or importance. The interference between memories explicitly depends on their strength, so that only if a stronger memory is acquired after the weaker one, then the weaker memory is erased. It is in this aspect that our model radically differs from those proposed in [12] and [17]. The process proposed has a clear functional interpretation of trying to keep important memories while discarding less important ones.

## The model

**Figure 1:**
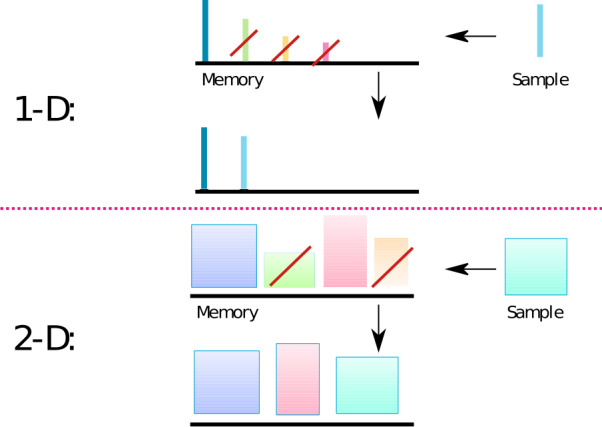
*Interference model of forgetting*. **1-D**. Each item is represented as a thin vertical bar. The height of the bar corresponds to the valence of an item. The top row bars above the black line represent items that are stored in memory just before the acquisition of a new item, shown on the right (Sample). All the items that have smaller valence (bar height) than the new item are discarded from memory (crossed by red bar), and the new item is added. Bottom row represents the memory content after the new memory is acquired. **2-D**. Same as 1-D, but each memory item has two valences represented by the width and height of a rectangular. In this case, all the items that have both valences smaller than the corresponding valences of the new item are discarded

To illustrate the main idea of our model, let us first consider a system that continuously acquires new memory items, each characterized by the scalar value *v* (valence), considered to be a measure of its importance and independently sampled from a distribution $P(v)$. The form of this distribution can be arbitrary, but we assume that it is not changing with time. For simplicity, we assume that memories are acquired at a constant rate (one new memory per time step). Each time a new item is sampled, it is stored in memory while all the previously stored items that have a smaller valence are discarded (“forgotten”; see Fig. 1, upper panel). Therefore the total number of stored items will increase if items with relatively small valences are sampled but can suddenly decrease if the sampled element is very potent. This process can be regarded as a crude approximation to retroactive interference. By the construction of the model, at any given moment the valence of the stored units will be an increasing function of their age, since units are retained only if following units have a smaller valence and are discarded otherwise. Therefore the probability that a unit will be forgotten at the next time step is a strictly decaying function of its age, that is, one of the most counterintuitive features of memory retention is inherently captured by the model. Mathematically, the retention function $R(t)$ is defined as the probability that a memory item is still retained in memory *t* time steps after its acquisition, which is equivalent to saying that it has a highest valence among $t+1$ memories (itself and *t* following ones). Since we assume that valences are independently sampled from the corresponding distribution, any one of them has the same chance of being the highest, and hence the retention function is given by

7 $$ R(t)=\frac{1}{t+1}. $$

We see that this simple model exhibits the uniform power-law scaling of memory retention for all times. It is important that there are no free parameters that affect the retention properties of the model, and, in particular, the form of the probability distribution $P(v)$ of valences has no effect on the model behavior. The $1/t$ scaling of the retention curve implies that the average number of memories does not saturate with time but continues to grow, which is an attractive feature of the model. However, if we compute the average number of items in memory after a long time *T* from the beginning of the acquisition process, then we obtain

8 $$ N(T)=\sum_{t=1}^{T} \frac{1}{t+1}\approx \log (T), $$

that is, the number of stored items is very small in relation to the total number of sampled ones. In particular, even after $T=10^{8}$ time units (several years of learning if one assumes a new memory acquisition per second), no more than twenty memories are retained, which is clearly not a reasonable estimate.

To address this problem, we considered two modifications of the model. In the first one, we assumed that rather than erasing all memories with smaller valence, the new memory only erases the one with the smallest valence, unless the new memory is itself the one with the smallest valence, in which case, none of the memories is erased. This model can be solved analytically as well (see Appendix B). In particular, the number of remaining memories grows linearly with time: $N(T) = \frac{T}{e}$, which seems to be unrealistic since it would predict a too rapid accumulation of memories. Here we present another generalization of the above model, which relaxes the unrealistic assumption about the single metric of importance for memories. Indeed, we can argue that each piece of acquired information may be very important in one context but trivial in another (see also Discussion below). This idea can be easily translated into the model by introducing a multidimensional valence distribution, where each component of the sample **v** represents its valence on a different domain. The forgetting rule in this case is expanded to all dimensions, and for an item to be forgotten, it is required that the newly acquired sample has a larger valence value on all axes (see Fig. 1, lower panel, for the two-dimensional case). The retention function in this extended model cannot be expressed in a closed form but can be iteratively computed with the following scheme:

9 $$ R_{n}(t) = \frac{1}{t+1}\sum _{k=1}^{t+1} R_{n-1}(k-1), $$

where *n* is the number of dimensions, and $R_{1}(t)$ is the retention curve of the one-dimensional model (equation (7)). To derive this equation inductively, consider a memory acquired at time 0 followed by *t* other memories. Let *k* be the rank of the original memory among the group of $t+1$ ones along the last valence dimension, that is, $k-1$ of the subsequently acquired *t* memories have higher valence along this dimension, whereas the rest have lower valence and hence cannot erase the original memory independently on other dimensions. For the original memory to survive for *t* time steps, it has to survive the $k-1$ potentially “dangerous” memories thanks to the first $n-1$ dimensions, the probability for this being $R_{n-1}(k-1)$ (definition of the retention function). Since all values of *k* from 1 to $t+1$ are equally likely and hence have a probability of $\frac{1}{t+1}$, the total retention probability, averaged over possible values of *k*, is given by equation (9).

Repeated application of equation (9) allows the exact calculation of the retention curve for arbitrary *n*. Assuming large *t*, this expression approximates to

10 $$ R_{n}(t) \approx \frac{1}{(n-1)!}\frac{\log ^{n-1}(t+1)}{(t+1)} $$

(see Appendix for the derivation), which has the same scaling as in the one-dimensional case (7) with logarithmic correction. This correction, aggregated over a long time *T*, leads to the total number of retained memories given by

11 $$ N_{n}(T) =\sum_{t=1}^{T} R_{n}(t) \approx \frac{1}{n!} \log ^{n}(T). $$

**Figure 2:**
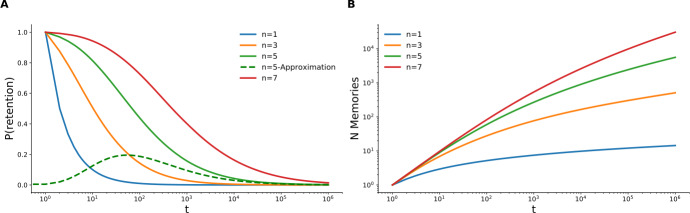
*Theoretical results*. **A**. Theoretical retention curves (9) for different number of valence dimensions *n*. The dashed green line shows the asymptotic approximation of equation (10) for $n=5$, which converges to an exact curve from $T \approx 10^{4}$. **B**. The average number of retained memories accumulated as a function of elapsed time from the beginning of acquisition

Figure 2 shows the plots for $R(t)$ and $N(T)$ for several values of dimensionality. For example, we see that for $n=5$, the number of retained memories after $T=10^{8}$ steps of acquisition is around few tens of thousands, which appears to be a reasonable estimate [18].

**Figure 3:**
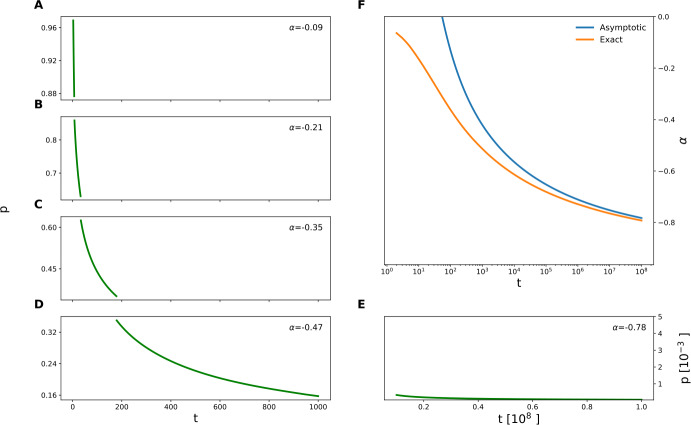
*Power fit of theoretical retention curves*. **A**–**E** Theoretical retention curve computed with equation (9) for $n=5$, plotted for different time windows. In the inset the estimated value of the power *α* for the corresponding window is shown. **F** The dependence of *α* on time (orange curve). The value of *α* very slowly approaches −1, so that even for $T=10^{8}$, it is still about −0.8. For comparison, the asymptotic estimate of *α* given by equation (14) is shown in blue

The above analysis shows that in the multidimensional case the retention curves deviate from simple power-law functions due to logarithmic corrections. We can still approximate the retention curve with a power-law function with a slowly changing exponent:

12 $$ R_{n}(t) \approx c(t) t^{\alpha (t)}, $$

where the exponent $\alpha (t)$ can be estimated as

13 $$ \alpha (t) = \frac{d(\log (R_{n}(t))}{d(\log (t))} $$

(see Fig. 3). We can see that the scaling exponent is slowly reduced to −1 for very large times, remaining significantly above that asymptotic value even for times as large as 10^8^. The asymptotic expression of the exponent can be derived by the asymptotic expression for the retention curve (equation (10)), resulting in

14 $$ \alpha (t) \approx -1 + \frac{n-1}{\log (t)}. $$

## Experiment

**Figure 4:**
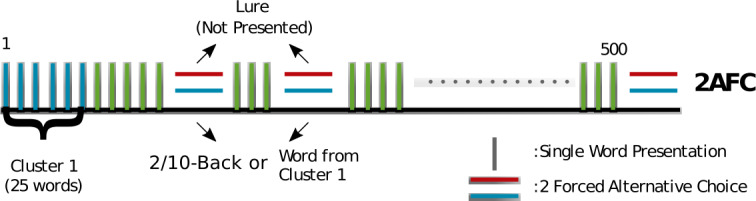
*Experimental protocol*. Vertical bars represent word presentations. Pairs of horizontal bars represent a delayed recognition task, where participants were presented with one word shown to them previously and one lure word. Participants were requested to click on the word they felt that they saw before. In total, 500 words were presented, and all first 25 words were queried at different moments. Additionally, participants undertook recognition tests for the second (25) and tenth (25) back word from the time of inquiry resulting in a total of 75 tests per participant. The 2/10 back conditions were conducted in blocks, i.e., no other queries were performed between presentation of the item to be queried and the query itself

**Figure 5:**
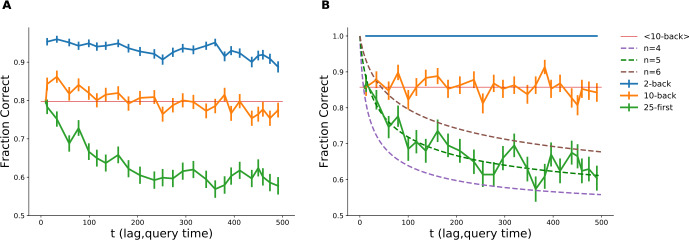
*Experimental results*. **A** Recognition performance for all participants who passed the qualification task (see Methods). **B** Recognition performance for participants that were perfect in the 2-back task. The experimental retention curve (recognition performance vs the lag between word presentation and delayed recognition task positions, solid green curve) declines for both groups. We can observe that performance in the 2-back task (solid blue curves, time here indicates the position of the recognition task) is declining with time, It is generally assumed that working memory capacity is 3–5 items, and therefore the drop of performance in the 2-back task indicates that a progressively smaller fraction of words was acquired toward the end of the trial due to fatigue, loss of attention, or other reasons. Selecting the group of participants (in panel **B**) that are perfect in the 2-back task, we ensured that all the words were acquired during presentation. For this group of participants, the performance in the 10-back task (orange solid curves, time indicates query position) remains constant throughout the experiment (compare with faint orange line representing mean performance in the 10-back task). The dashed lines represent theoretical retention curves computed with equation (10) corrected for guessing (see Methods), for different number of dimensions *n*

To test whether the model conforms with human memory performance and to estimate the number of dimensions for the valence distribution of memory, we designed an experimental protocol based on the two-alternative-forced-choice delayed recognition task [19]. The experiment was performed on Amazon’s Mechanical Turk® platform. Participants were presented with a sequence of 500 words, intermittent with recognition attempts. During recognition inquiries, participants were prompted to select between two words on the basis of which word they remembered as having previously appeared: one choice constituted a word presented earlier in the sequence (either 2 or 10 words before the recognition attempt or one of the first 25 presented) and another one a lure (see Fig. 4). Following [19], we make the simplifying assumption that if a previously presented word is still in memory, then the participant will provide a correct answer; otherwise, the response is going to stem from guessing. The experimental results are shown in Fig. 5. Figure 5A shows the results for all 471 participants in the experiment. We can observe that the probability to recognize a word decays toward the chance level (50% correct recognition responses) as a function of lag between presentation and inquiry (green line). The probability of recognizing the word presented 10 (10-back task) or 2 (2-back task) positions before the recognition prompt also declines as the experiment furthers in time (blue and orange lines). This can result from either proactive interference (when previously memorized items interfere with an acquisition of new words) or general fatigue accompanied with diminished attention, leading to disruption of new word acquisition. Since the short-term memory capacity is estimated to be 3–5 items [20], we conjectured that the last two words, if acquired, should stay in short-term memory. We therefore selected 197 participants who exhibited perfect performance on the 2-back recognition task (see Fig. 5B). Indeed, these participants show no decline of performance for the 10-back test either, indicating the absence of forward interference. Their retention performance (green line) is in agreement with the theoretical prediction for $n=5$ (dashed green line).

## Discussion

We proposed a phenomenological model of forgetting that is broadly compatible with retention curves reported in the earlier literature and with focused recognition experiments performed specifically for this study. The main idea of the model is strength-dependent retroactive interference between the memories, so that only if a stronger memory is acquired after the weaker one, then the weaker one is erased. The model results in power-law retention curves with exponents that very slowly decline toward −1, remaining significantly above this asymptotic value for all realistic time lags that can be measured experimentally. The model is founded on a single computational principle that has a clear functional meaning; namely, we assume that the system tries to maintain important memories at the expense of less important ones, and to this end, each newly acquired element erases already stored ones that are less significant. Importance is evaluated by a multidimensional valence measure such that memories that remain are characterized by relatively higher valence measures in one or more dimensions. The number of dimensions is a free parameter of the model and was estimated to be 5 based on our recognition experiments with long randomly assembled lists of words. The nature of these differential valence dimensions is not specified in the model. For example, the memory of an event could have five domains (who, when, where, what, and why), each of them defining a different axis of importance. If that event involves a very relevant person (therefore a high value on the “who” axis), then it would be likely to be retained in memory, even if what happened was relatively insignificant. Another way to view a piece of information as a multidimensional element comes from the work on semantic representations of words. In particular, it has been shown that the same word pertaining to different conceptual groups activates different parts of the brain according to the contextual associations made upon acquisition [21].

Similarly to [12], the average life-span of memories in the model diverges due to accumulation of very strong memories, and hence the process never reaches a steady state with the number of memories increasing, albeit with decreasing speed. Besides the number of dimensions, the model has no single free parameter, and hence the observation that it fits the experimental results so well is quite surprising. It shows that retroactive interference, which is well documented in psychological studies [16], is by itself sufficient to account for realistic forms of memory retention. Consolidation is thus not critical for this property of memory, which does not preclude its role in other aspects of memory not addressed in this study.

Several of the assumptions of the model clearly oversimplify the memory system and do not appear realistic (some of them are mentioned in Introduction). In particular, the possible neuronal mechanism for erasure of previously acquired memories with lower valence in each dimension is not clear. Moreover, the valence of each memory is supposed to be stable for the duration of memory, and the distribution of valences not constrained in the model is supposed to be stationary. In real life, we could imagine that some memories’ importance could be altered in time, whereas the distribution of new memory valences could also potentially change, for example, due to aging or other life changes. It would be interesting to consider how the system would adapt to these changes by slowly replacing memories that become less relevant by the more relevant ones. More generally, it will be of critical importance to consider the generality of the model to different types of memory, such as traumatic experiences for which consolidation and reconsolidation could play an important role. Our recognition experiments indicate that a five-dimensional version of the model predicts well the retention curve measured with recognition experiments using randomly assembled lists of words and one particular presentation speed. It remains to be seen whether the same number of dimensions will describe other presentation protocols and other types of material, such as images, short sentences, and so on.

The simplicity of the model obviously does not guarantee its validity because other models, based on different principles, can also account for the same observations. We should therefore focus on critical predictions that could potentially distinguish our model from the previous ones. One such critical prediction of the current model concerns the role of ordering in memory. From a mathematical point of view, memories in the model can be considered as a partially ordered set (see e.g. [22]), that is, a memory characterized by a particular set of values in all dimensions erases another memory with lower values in all dimensions, but any two memories that do not have consistent relation between the values (e.g. two memories that exhibit opposite relation between the values in two dimensions) cannot erase each other. In other words, some memory pairs can be ordered, whereas other pairs cannot. The “linear extension of the partial order” theorem states that memories can be reordered so that for all memory pairs with consistent relation between them, the erasing memory is placed before the one it erases. For this ordering, the model predicts that none of the memories will be erased, that is, all they will remain intact after the list is presented. This feature of our model is highly nontrivial and does not hold for the models of [12, 17]. Finding the best presentation order is challenging because it would require knowledge of memory values that are not known and could well be individual to different people, but we are developing experimental approaches that could circumvent this problem. It is interesting that if this prediction is confirmed in future experiments (even if only partially), then this will also confirm that power-law forgetting is a genuine phenomenon and not an artifact of averaging over subjects, because the optimal presentation order is most probably individual to each subject. This kind of predictions, if properly tested, could hopefully encourage the development of new experimental paradigms, which could shed light on the true mechanisms of forgetting.

## Methods

### Participants, stimuli, and procedure

A total of 900 participants were recruited to undertake a series of recognition tasks, designed to be performed utilizing Amazon’s Mechanical Turk® (mTurk) platform (https://www.mturk.com). Ethics approval was obtained by the Institutional Review Board of the Weizmann Institute of Science, and each participant accepted an informed consent form before participation. Participants were first required to complete the qualification task, and if they met criteria described below, then they were allowed to participate in the main experiment (471 people). Participation was compensated at 10 cents for the qualification task and 30 cents for the regular task.

#### Delayed recognition tasks

All tasks performed in this study were two-alternative forced choice delayed recognition tasks. Experiments were initiated with participants clicking on a “Start Experiment” button. A stream of words was presented sequentially utilizing the standard interface on mTurk’s website for Human Intelligence Tasks, using a custom HTML file with embedded Javascript. Each word was briefly flashed for a duration of 1 s followed by a blank screen of 0.5 s. The words were displayed centrally on a white screen in black font. At random points during the trial and once in the end, after all words were presented, the presentation of words paused, and participants were given a choice of two words in the form of vertically aligned buttons. Each button was randomly assigned with a word, one that was previously presented during the trial and one new word (lure). The participants were instructed to select the button containing the word they remembered seeing. After the selection, presentation resumed automatically. The list of presented words for each participant was randomly generated by sampling without replacement from a pool of 751 words, which was produced by selecting English words [23] that exhibited a frequency larger than ten per million [24]. Each participant performed only one qualification and one main task trials.

#### Qualification

Our previous experience on the mTurk platform showed that many workers are poorly performing and are not following the experiment instructions carefully. Therefore each participant was first presented with a simpler and shorter task. A recognition delayed task with one hundred words in a stream was presented to participants. In 25 recognition tasks the participants were questioned about the word presented just before the last one (2-back task). We reasoned that two last presented words should stay in short-term memory if participants are attending to stimuli and following instruction. Therefore we informed people who performed the qualification task with a success rate of more than 95% that they may perform the main experiment. The rest were compensated for participation in qualification experiment.

#### Main task

Similarly to the qualification task, the participants had to attend to a stream of words, in this case, five hundred in total. During the trial, at seventy four random points (excluding the first 25 words) plus at the end of the list, they were prompted for a delayed recognition of a previously shown word versus a lure word. Twenty five of them requested a recognition of the second-back word as in the qualification, twenty five for the tenth-back, and twenty five for the first twenty five words presented. Recognition tasks were randomly intermixed.

### Analysis

In Fig. 5 the lag was computed as the difference between a query position and a presentation position in the stream of words. For example, if before the 100th word, there was a recognition task related to 15th word the lag is 85. In the figure the mean fraction of correct recognition is shown for lag bins with equal population of measurements (197) per bin, averaged across all participants having questions with query lags inside the bin. Not all participants had queries for all bins.

#### Correction for guessing

In computing the theoretical performance for the recognition task, we assumed that if a person is remembering the presented word, then she/he would correctly point out to the presented word. In the case where participants do not remember the word, we assume that they are guessing and therefore choosing with equal probability. Therefore we may express the recognition performance as

15 $$ p(t) = R(t) + \frac{1}{2}\bigl(1-R(t)\bigr) = \frac{1+R(t)}{2}, $$

where $p(t)$ is the fraction of correct responses in recognition task plotted in Fig. 5B (dashed curves), and $R(t)$ is the retention probability of a memory acquired *t* time steps before testing.

## Figures and Tables

**Figure 6:**
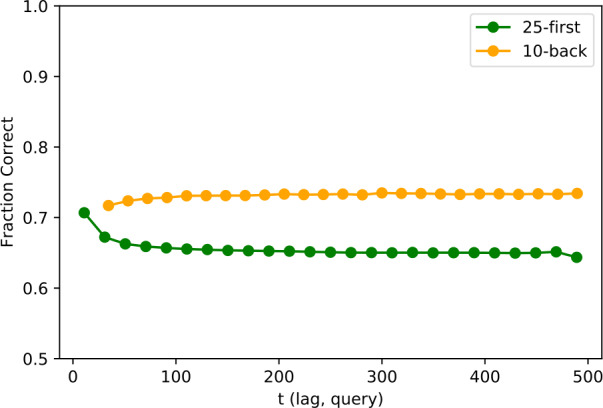
*Alternative model simulations of the recognition experiment*. Simulation of 100000 runs of the recognition experiment presented above using the model with the modified erasure rule. The green curve corresponds to the first-25 task and the orange one to the 10-back task

## Data Availability

The datasets used and/or analyzed during the current study are available from the corresponding author on reasonable request.

## Appendix A: Solution of Kahana–Adler model

We analyze the version of Kahana–Adler model [12] with linear decay of memory strength $S(t)=a-bt$ with positive random coefficients *a* and *b*. Other types of passive decay produce similar results. For simplicity, we assume that memory is forgotten when its strength dips below zero. The probability that a memory is still available at time *t* after its inception is given by

16 $$ R(t)=Prob(a-bt>0)= \int _{0}^{\infty }db P_{b}(b) \int _{bt}^{\infty }da P_{a}(a), $$

where $P_{a}$ and $P_{b}$ are the probability densities of *a* and *b*, respectively. Introducing the new variable $b \rightarrow bt $ and taking the limit $t \gg 1$, we obtain

$$\begin{aligned} R(t)&=\frac{1}{t} \int _{0}^{\infty }db P_{b}(b/t) \int _{b}^{\infty }da P_{a}(a) \\ &\approx \frac{P_{b}(0)}{t} \int _{0}^{\infty }db \int _{b}^{\infty }da P_{a}(a) \\ &=\frac{p_{b}(0)}{t}\langle a\rangle , \end{aligned}$$

where the third line is obtained by integration by parts of the previous line, and $\langle a\rangle$ stands for the average value of *a*. Finally, we note that the $1/t$ scaling of retention implies that the probability density of the life-span of a memory $P_{\mathrm{life}}(t)$ scales as $1/t^{2}$ asymptotically for large *t*:

17 $$ P_{\mathrm{life}}(t)=-\frac{d}{dt}R(t)\sim \frac{1}{t^{2}}, $$

which it turn implies that the average memory life-span is infinite.

## Appendix B: Solution of the retrograde interference model with modified erasure rule

As mentioned in the paper, we consider a version of the retrograde interference model where the weakest memory is erased upon the acquisition of the new one, unless the new memory is itself the weakest of all that are currently remaining. It turns out that asymptotically there is a sharp threshold for memory strength, such that above the threshold, all memories (except for a finite number) remain in the system indefinitely, whereas below the threshold, all memories are eventually erased. The value of the threshold and hence the accumulation rate of memories can be calculated as follows. Without loss of generality, assume that the memory strengths are uniformly distributed in the interval between 0 and 1. Denote the threshold as *θ* ($0<\theta <1$). Each memory below threshold (BTM) is eventually erased upon presentation of another memory, which itself could be either above (ATM) or below threshold. Denote by *p* the fraction of BTMs that are erased by the presentation of one of ATMs. Since each ATM erases exactly one BTM, and all ATMs remain in memory, we get

18 $$ p = \frac{1-\theta }{\theta }. $$

On the other hand, for each BTM with strength *x* ($x<\theta $), the probability that it is erased by one of BTMs is $\frac{1-\theta }{1-x}$, and hence *p* can also be obtained by averaging this probability over all *x* between 0 and *θ*:

19 $$ p = \frac{1}{\theta } \int _{0}^{\theta } \frac{1-\theta }{1-x} = \frac{1-\theta }{\theta } \log \frac{1}{1-\theta }. $$

From the last two equations we obtain $\theta = 1 - \frac{1}{e}$, which in turn implies that the number of memories that remain after time *T* is $\frac{T}{e}$.

We also simulated 100,000 runs of our recognition experiment under this model, and the results can be seen in Fig. 6. The green curve represents the average recognition performance for the first-25 task. The orange curve shows the results of the 10-back task. Comparing these simulations to the experimental results shown in Fig. 5, we see that the model does not match the data well; in particular, the retention curve exhibits shape very different from the experimental one.

## Appendix C: Multidimensional retention function asymptotic behavior

We use the iterative equation (9) to derive the asymptotic behavior of the retention function $R_{n}(t)$ in the limit of large *t*. To this end, we guess the functional form of $R_{n}(t)$ in this limit as

20 $$\begin{aligned} \begin{aligned} &R_{n}(t)= a_{n} \frac{ \log ^{b_{n}}(t+1) }{t+1}, \\ &a_{1}= 1, \\ &b_{1}= 0. \end{aligned} \end{aligned}$$

Substituting this ansatz into equation (9) and approximating sums over time by the corresponding integrals, we obtained the following iterative equations for the factors $a_{n}$ and $b_{n}$:

21 $$\begin{aligned} &a_{n}=\frac{a_{n-1}}{b_{n}}, \end{aligned}$$

22 $$\begin{aligned} &b_{n}=b_{n-1}+1, \end{aligned}$$

which have an obvious solution of $a_{n}=\frac{1}{(n-1)!}$, $b_{n}=n-1$, resulting in equation (10).
